# Do women spend longer on wait lists for coronary bypass surgery? Analysis of a population-based registry in British Columbia, Canada

**DOI:** 10.1186/1471-2261-7-24

**Published:** 2007-08-02

**Authors:** Adrian R Levy, Boris G Sobolev, Lisa Kuramoto, Robert Hayden, Stuart M MacLeod

**Affiliations:** 1 Department of Health Care and Epidemiology, University of British Columbia (BC), Vancouver, Canada; 2 Centre for Health Evaluation & Outcome Sciences, Providence Health Care, Vancouver, Canada; 3 Centre for Clinical Epidemiology and Evaluation, Vancouver General Hospital, Vancouver, Canada; 4 Department of Surgery, Royal Columbian Hospital, New Westminster, Canada; 5 BC Cardiac Registries, Provincial Health Services Authority, Vancouver, Canada; 6 BC Research Institute of Women's and Children's Health, Vancouver, BC, Canada; 7 BC Provincial Health Services Authority, Vancouver, Canada

## Abstract

**Background:**

Studies have shown patients who are delayed for surgical cardiac revascularization are faced with increased risks of symptom deterioration and death. This could explain the observation that operative mortality among persons undergoing coronary artery bypass surgery (CABG) is higher among women than men. However, in jurisdictions that employ priority wait lists to manage access to elective cardiac surgery, there is little information on whether women wait longer than men for CABG. It is therefore difficult to ascertain whether higher operative mortality among women is due to biological differences or to delayed access to elective CABG.

**Methods:**

Using records from a population-based registry, we compared the wait-list time between women and men in British Columbia (BC) between 1990 and 2000. We compared the number of weeks from registration to surgery for equal proportions of women and men, after adjusting for priority, comorbidity and age.

**Results:**

In BC in the 1990s, 9,167 patients aged 40 years and over were registered on wait lists for CABG and spent a total of 136,071 person-weeks waiting. At the time of registration for CABG, women were more likely to have a comorbid condition than men. We found little evidence to suggest that women waited longer than men for CABG after registration, after adjusting for comorbidity and age, either overall or within three priority groups.

**Conclusion:**

Our findings support the hypothesis that higher operative mortality during elective CABG operations observed among women is not due to longer delays for the procedure.

## Background

In publicly funded health care systems, priority wait lists are commonly used to manage access to elective cardiac surgery [1]. While queuing according to urgency of intervention is designed to facilitate access to surgery within a clinically appropriate time [2], patients who are delayed for surgical cardiac revascularization are faced with increased risks of worsening symptoms [3] and death [4,5]. The additional risks incurred by longer delays may be of particular concern for women with cardiovascular disease because, at presentation, women are more likely than men to have comorbid medical conditions such as hypertension, diabetes or obesity [6-11]. These comorbid medical conditions may increase the amount of time that women wait for CABG.

A number of studies have shown that the operative mortality among women undergoing CABG surgery is higher than that of men [12,13]. However, there is no information on whether women wait longer than men for CABG, after adjusting for age, severity of disease and comorbidity. It is therefore difficult to ascertain whether higher operative mortality may be due to delayed access to care in the pre-surgical period. Addressing this question is important for improving the care of persons with cardiovascular disease because, if differences are due to longer waiting times, it might be feasible to implement strategies to reduce waiting times among women.

The objective of this study was to compare the time from registration on the wait list to CABG between women and men, after adjusting for differences in age, severity of coronary artery disease, and comorbidity. To examine the consistency over time of any effects of sex on waiting times, comparisons were done across synthetic cohorts of patients defined by two-year periods of registration on the wait lists: 1991–92, 1993–94, 1995–96, 1997–98, or 1999–2000.

## Methods

### Data sources

The BC Cardiac Registries (BCCR) prospectively capture the occurrence and timing of registration, surgery, or removal from the wait lists without surgery, for all patients accepted for cardiac surgery procedures in the four heart-surgery centers delivering services in BC (population of four million) [14]. Registered patients were removed from the wait lists without surgery if they died, declined the operation, accepted surgery from another surgeon, moved away, or continued with medical management. Cardiac surgeons in BC have developed common guidelines for prioritizing patients and assigning the suggested waiting time for surgery based on angina symptoms, affected coronary anatomy, non-invasive test results, and left ventricular function impairment as described elsewhere [15]. Using those guidelines, each patient was classified by the surgeon into one of the following three groups: priority 1 if the suggested time to surgery was three days, priority 2 if the suggested time to surgery was six weeks, and priority 3 if the suggested time to surgery was 12 weeks.

### Patients

There were 9,366 records of registration for isolated CABG added to the Registry between January 1991 and December 2000. We excluded 135 records of patients who were: emergency cases (30), removed on the registration date (101), and had missing operating room reports (4). All remaining 9,231 records had either the surgery date or the date and reason of removal from the list without surgery. We restricted the analyses to the first 52 weeks after registration so that 475 (5%) patients remaining on the lists at 12 months were censored. Of those, 167 eventually underwent surgery; seven died; 78 received medical treatment; 104 declined surgery; 17 were transferred to another surgeon or hospital; and 102 were removed for other reasons.

### Comorbidity

To control for co-existing conditions, each patient was classified as (1) presenting with no co-existing conditions, (2) presenting with a major comorbid condition including congestive heart failure, diabetes, chronic obstructive pulmonary disease, cancer, or rheumatoid arthritis, or (3) presenting with a minor comorbid condition including other coexisting chronic conditions including peripheral vascular disease, cerebrovascular disease, dementia, peptic ulcer disease, hemiplegia, renal disease, or liver disease [16]. The first set of conditions were those originally used in a study on the appropriateness of coronary revascularization [17]. As we were concerned that there were other potential concomitant illnesses that could delay surgery, we added the other category that included conditions from Charlson comorbidity index [18]. We entered two indicator variables in the models to represent the three comorbidity categories.

### Statistical methods

Waiting times were analyzed as prospective observations beginning at the time of registration. Each patient had a waiting time calculated in calendar weeks from registration to surgery or removal for other reasons. The cumulative probability of undergoing surgery as a function of waiting time was estimated using the Kaplan-Meier method [19]. Patients removed from the list for reasons other than surgery were treated as censored observations.

Primary comparisons were done across synthetic cohorts of patients defined by two-year periods of registration on the wait lists. Within each registration period, differences in the distributions of wait-list times between women and men were examined using the log rank-test [20].

The effect size for each period was estimated using hazard ratios for surgery derived from a Cox proportional hazards model [21] in which we stratified on age. The priority group and the comorbidity measures were included as independent variables in the Cox model to estimate adjusted effects. Hazard ratios (HR) for women evaluate the conditional probability of undergoing CABG relative to men at any week on the list. The weekly surgery rate was calculated by dividing the number of operations by the total number of patient-weeks on the list.

The Clinical Research Ethics Board of the University of British Columbia approved the study protocol in September, 2001. Individual consent was waived.

## Results and discussion

In BC in the 1990s, 9,167 patients aged 40 years and over were registered on wait lists for CABG and spent a total of 136,071 person-weeks waiting. Of 9,167 persons aged 40 to 89 y who were registered for CABG in BC between 1991 and 2000, about 18% (1,629) were women (Table 1). At registration for CABG, among women, 19% were under age 60 y and 42% were over age 70 y, compared with 31% of men under age 60 y and 30% over age 70 y. There was a significant difference in the distribution of age between women and men (X^2 ^= 128.3, df = 4, P < 0.0001). The number of women registered ranged from a low of 297 in 1993–1994 to a high of 379 in 1995–1996 (data not shown).

**Table 1:** Characteristics of 9,167 patients registered for CABG surgery in British Columbia, 1991–2000

	Women (N = 1,629)		Men (N = 7,538)	
	
Characteristic	N	(%)	N	(%)
Age Group (y)				
40–49	68	(4.2)	600	(8.0)
50–59	241	(14.8)	1764	(23.4)
60–69	638	(39.2)	2892	(38.4)
70–79	633	(38.9)	2137	(28.3)
80–89	49	(3.0)	145	(1.9)
Urgency at Registration				
Priority 1	123	(7.6)	534	(7.1)
Priority 2	1116	(68.5)	5339	(70.8)
Priority 3	377	(23.1)	1567	(20.8)
Unknown	13	(0.8)	98	(1.3)
Comorbidity at Registration				
No comorbidity	750	(46.0)	3985	(52.9)
Major comorbidity (CHF, diabetes, COPD, rheumatism, cancer)	463	(28.4)	1540	(20.4)
Other conditions	416	(25.2)	2013	(26.7)
Registration Period				
1991–1992	318	(19.5)	1394	(18.5)
1993–1994	297	(18.2)	1574	(20.9)
1995–1996	379	(23.3)	1619	(21.5)
1997–1998	350	(21.5)	1527	(20.3)
1999–2000	285	(17.5)	1424	(18.9)

Abbreviations: CABG = outpatient isolated coronary artery bypass surgery; CHF = congestive heart failure; COPD = chronic obstructive pulmonary disease

At registration, 46% of women had no comorbid conditions recorded, 25% had at least one minor comorbid medical condition and 28% had at least one major comorbid condition. Women were 20% less likely than men to have no comorbid conditions (OR = 0.8) and 50% more likely to have a major comorbid condition (OR = 1.5). There was a significant difference in the distribution of comorbid medical conditions between women and men (X^2 ^= 51.9, df = 2, P < 0.0001). A similar proportion of women and men received the operation within the recommended waiting time (Table 2).

**Table 2:** Outcomes of registration for CABG surgery in British Columbia 1991–2000 at 52 weeks

	Women (N = 1629)		Men (N = 7538)	
	
Outcomes	N	(%)	N	(%)
Underwent Surgery*				
Within recommended time	534	(32.8)	2393	(31.7)
Beyond recommended time	845	(51.9)	4120	(54.7)
Between 1 and 12 weeks, priority unknown	8	(0.5)	33	(0.4)
Removed without surgery**				
Died while waiting	9	(0.6)	81	(1.1)
Medical treatment	46	(2.8)	128	(1.7)
Patient request	42	(2.6)	145	(1.9)
Transferred or moved	12	(0.7)	87	(1.2)
Other reason	45	(2.8)	166	(2.2)
Still on wait list at 52 weeks	88	(5.4)	385	(5.1)

Abbreviation: CABG = outpatient isolated coronary artery bypass surgery

* χ^2 ^= 2.0, df = 2, p = 0.36

** χ^2 ^= 14.8, df = 4, p = 0.0052

Over all periods, at registration for CABG, approximately equal proportions of women and men were in priority group 1 (OR = 1.0, 95% CI: 0.8, 1.2 (adjusted for year and age)), a lower proportion of women was in priority group 2 (OR = 0.9, 95% CI: 0.8, 1.0 (adjusted for year and age)), and a higher proportion was in priority group 3 (OR = 1.2, 95% CI 1.0, 1.3 (adjusted for year and age)) (Table 3). There was a significant difference in the distribution of priority group among women and men (X^2 ^= 7.72, df = 3, P = 0.0521).

**Table 3:** Distribution of patients registered for CABG in British Columbia 1991–2000 by priority group and registration period

	Priority 1		Priority 2		Priority 3	
	
Registration Period	N	(%)	N	(%)	N	(%)
	Women					

1991–1992	27	(8.5)	224	(70.4)	65	(20.4)
1993–1994	21	(7.1)	210	(70.7)	64	(21.5)
1995–1996	47	(12.4)	248	(65.4)	81	(21.4)
1997–1998	25	(7.1)	238	(68.0)	83	(23.7)
1999–2000	3	(1.1)	196	(68.8)	84	(29.5)
All periods*	123	(7.6)	1116	(68.5)	377	(23.1)

	Men					

1991–1992	88	(6.3)	990	(71.0)	267	(19.2)
1993–1994	89	(5.7)	1160	(73.7)	317	(20.1)
1995–1996	202	(12.5)	1105	(68.3)	291	(18.0)
1997–1998	91	(6.0)	1083	(70.9)	341	(22.3)
1999–2000	64	(4.5)	1001	(70.3)	351	(24.6)
All periods**	534	(7.1)	5339	(70.8)	1567	(20.8)

Abbreviation: CABG = outpatient isolated coronary artery bypass surgery

*Excludes 13 patients with unknown priority

**Excludes 98 patients with unknown priority

Among women, the distribution of priority group at registration for CABG remained approximately constant during the first four periods; in the final period, there was a reduced proportion in priority group 1 and an increased proportion in the priority group 3. A similar pattern was observed among men, with a relatively constant distribution of priority groups during the first four periods and a shift in the last period to a reduced proportion in the most urgent priority group and an increased proportion in the least urgent priority group.

Among women and over all registration periods, the median wait list time for outpatient isolated CABG in BC was 11 weeks, ranging from 7 weeks in the earliest period to 16 weeks in the middle period (Table 4). Among men and over all registration periods, the median wait list time was also 11 weeks, ranging from 9 weeks in the earliest period to 14 weeks in the middle period. As measured by the interquartile range (IQR), the variability in wait list time over all periods was 1 week longer for women than men (18 weeks versus 17 weeks).

**Table 4:** Percentiles of wait-list time (weeks) for women and men registered for CABG in British Columbia 1991–2000 by registration period

	Percentile					
	
Registration Period	10th	25th	50th	75th	90th	90th – 50th
	Women					

1991–1992	1	2	7	19	43	36
1993–1994	1	4	8	18	49	41
1995–1996	1	6	16	27	53	37
1997–1998	3	6	15	26	40	25
1999–2000	3	6	11	19	47	36
All periods	1	5	11	23	49	38

	Men					

1991–1992	1	3	9	19	44	25
1993–1994	2	4	9	18	44	25
1995–1996	1	6	14	26	46	32
1997–1998	2	6	13	24	43	30
1999–2000	3	6	10	19	39	29
All periods	2	5	11	22	43	32

Abbreviation: CABG = outpatient isolated coronary artery bypass surgery

One difference between women and men was observed for those patients with longer waiting times: the wait-list interval requiring 40% of operations in patients staying on the lists longer than the median time was 6 weeks longer among women than men, as measured by the differences between 90^th ^and 50^th ^percentiles. The differential between women and men reached a maximum 14 weeks in 1993–1994.

**Figure 1:**
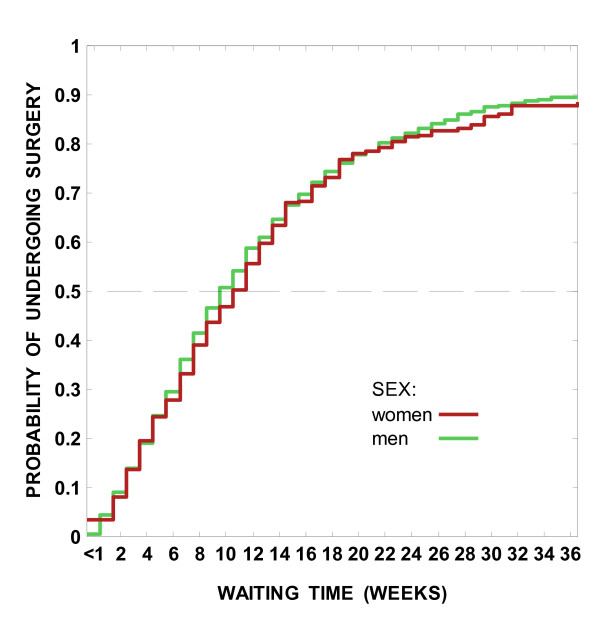
Characteristics of 9,167 patients registered for outpatient isolated coronary artery bypass surgery in British Columbia, 1991–2000.

The differences in time spent on the wait lists were not significantly different between women and men in any calendar period of registration (P > 0.10 for all periods). The distributions of the estimated probability of undergoing CABG at each week on the wait list for the period 1999–2000 were overlapping for women and men over the first 36 weeks after registration (Figure 1).

For women, the average weekly rate of operations per 100 varied from 6.9 (6.1–7.7) in the 1991–1992 cohort to 4.7 (4.2–5.2) in the 1995–1996 cohort to 5.8 (5.1–6.6) in the 1999–2000 cohort (Table 5). At any week on the list, the conditional probability of undergoing surgery was reduced by 39% in 1995–1996 women, HR = 0.61 (0.51–0.72), and by 13% in 1999–2000 women, HR = 0.87 (0.73–1.05), relative to the period 1991–1992, after adjusting for priority, comorbidity, and age.

**Table 5:** Average weekly rate of CABG in British Columbia, 1991–2000 and adjusted hazard ratios by registration period

Registration Period	Number of operations	Total waiting time, weeks	Crude Rate, per 100	SE	RR	95% CI*
	Women					

1991–1992	281	4094.0	6.9	(0.4)	1.00	Referent
1993–1994	260	3892.5	6.7	(0.4)	0.98	(0.82, 1.17)
1995–1996	313	6676.5	4.7	(0.3)	0.61	(0.51, 0.72)
1997–1998	293	5587.5	5.2	(0.3)	0.68	(0.57, 0.81)
1999–2000	240	4103.5	5.8	(0.4)	0.87	(0.73, 1.05)

	Men					

1991–1992	1211	18773.0	6.5	(0.2)	1.00	Referent
1993–1994	1370	21221.0	6.5	(0.2)	1.01	(0.93, 1.09)
1995–1996	1403	27353.0	5.1	(0.1)	0.72	(0.66, 0.78)
1997–1998	1311	24570.0	5.3	(0.1)	0.79	(0.72, 0.85)
1999–2000	1251	19799.5	6.3	(0.2)	0.93	(0.86, 1.01)

Abbreviations: CABG = outpatient isolated coronary artery bypass surgery; SE = standard error; HR = hazard ratio; 95% CI = 95% confidence interval

*adjusted for priority group and comorbidity; stratified by age.

0 patients were on the wait list on December 31, 2001

For men, the average weekly rate of operations per 100 varied from 6.5 (6.1–6.8) in the 1991–1992 cohort to 5.1 (4.9–5.4) in the 1995–1996 cohort to 6.3 (6.0–6.7) in the 1999–2000 cohort. At any week on the list, the conditional probability of undergoing surgery was reduced by 28% in 1995–1996 men, HR = 0.72 (0.66–0.78), and by 7% in 1999–2000 men, HR = 0.93 (0.86–1.01), relative to the period 1991–1992, after adjusting for priority, comorbidity, and age.

Within priority groups, there was little evidence that women waited longer than men for CABG after registration, after adjusting for comorbidity, age and sex (Table 6).

**Table 6:** Rate Ratios* by sex and priority group for patients undergoing CABG in British Columbia 1991–2000

	Priority 1		Priority 2		Priority 3	
	
Sex	RR	95% CI	RR	95% CI	RR	95% CI
Men	1.00	Referent	1.00	Referent	1.00	Referent
Women	0.88	(0.69, 1.14)	0.99	(0.92, 1.06)	1.01	(0.88, 1.16)

Abbreviations: CABG = outpatient isolated coronary artery bypass surgery; RR = rate ratio; 95% CI = 95% confidence interval

*adjusted for comorbidity; stratified by age and registration period

## Conclusion

In this paper we found little evidence that women waited longer than men for elective CABG in BC during the 1990s. After adjusting for comorbidity and age, the time spent on CABG wait lists did not differ between women and men in any calendar period of registration or within any of the three priority groups. A similar proportion of women and men received the operation within the recommended waiting time.

The distribution of women undergoing CABG was lower than reported in other jurisdictions [6,8,22,23] and within the range reported in other Canadian provinces: a lower proportion (12%) of women was observed in Nova Scotia [3] and a higher proportion (30%) in Alberta [24].

The significance of our findings can be understood within the context that women have higher mortality after CABG [6,25-28]. This issue was addressed using the United States Society of Thoracic Surgeons National Cardiac Surgery Database to examine peri-operative survival among 344,913 patients undergoing CABG between 1994 and 1997 [6]. After adjustment for other risk factors, female sex remained an independent predictor of operative mortality in all but very high risk patients. The main finding in the current study – that waiting times did not differ between women and men in BC – supports the hypothesis that the higher operative mortality among women is due to biological differences. Studies from the Cleveland Clinic and the Northern New England Study Group have shown the impact of body size on peri-operative CABG mortality [29,30]. Women typically have a smaller body surface area than men which in turn is associated with smaller hearts and correspondingly diminutive coronary arteries. This is thought to increase the technical difficulty of CABG and contribute to poorer outcome [31]. Alternatively, the differences in operative mortality may be due to a delay in treatment or in referral to catheterization [32]. Other Canadian investigators found that the median duration between cardiac catheterization and surgery did differ between women and men waiting for CABG or aortic valve replacement in Nova Scotia [3].

We also found that, for one portion of the waiting time distribution that included patients who waited longer than the median waiting time as measured by the differences between 90^th ^and 50^th ^percentiles, the waiting time was six weeks longer among women than men. An explanation of this finding remains speculative and could serve as the basis of a future study.

As reported by other investigators [6-11], we found that women were significantly older and more likely to have a major comorbid medical conditions when registered for CABG. Part of this difference stems from women presenting with initial symptoms of cardiovascular disease at an older age. This may also be indicative of differences in health seeking behavior by women [33,34] or in referral patterns from a cardiologist to a cardiac surgeon for women with symptoms of cardiovascular disease [32].

### Limitations

The internal validity of this study was high and it is unlikely that potential biases could have materially affected the results. Selection bias was minimized because the registry maintains a record for virtually every person assessed by a cardiac surgeon in BC and active follow-up is undertaken for all persons registered. Information bias was not a concern because there is no reason to believe that any coding or other errors occurred differentially among women and men. While there is always the possibility of confounding in an observational study, the likelihood of a major unknown confounder is small because it would need to have exerted a strong influence to substantially affecting the interpretation and results, and we are unaware of any powerful factor that affected waiting time.

The main question regarding the validity of the study is the generalizability of the results. A study examining whether the waiting time for CABG differs between women and men in another jurisdiction that employs priority wait lists for CABG would be valuable. It would also be of considerable interest to determine the impact of sex in health systems that use wait lists but have different types of reimbursement mechanisms.

## Competing interests

The author(s) declare that they have no competing interests.

## Authors' contributions

ARL conceived and designed the study, acquired the data, interpreted the results, and drafted the manuscript. BGS conceived and designed the study, analysed the data, interpreted the results, and drafted the manuscript. LK analysed the data and interpreted the results. RH participated in the design of the study, helped acquire the data, and interpreted the results. SMM conceived and designed the study and interpreted the results. All authors read and approved the final manuscript.

## Pre-publication history

The pre-publication history for this paper can be accessed here:


